# Research on the molecular mechanisms and therapeutic strategies of reactive oxygen species in nasopharyngeal carcinoma

**DOI:** 10.3389/fphar.2026.1980464

**Published:** 2026-10-16

**Authors:** Yuting Gu, Bi Qiang

**Affiliations:** Department of Otolaryngology Head and Neck Surgery, The First Affiliated Hospital of Yangtze University, The First People’s Hospital of Jingzhou, Jingzhou, Hubei, China

**Keywords:** anti-tumor therapy, epstein-barr virus, nasopharyngeal carcinoma, oxidative stress, programmed cell death, reactive oxygen species, redox homeostasis

## Abstract

Nasopharyngeal carcinoma (NPC) exhibits significant geographical clustering and etiological heterogeneity. It is particularly prevalent in Southeast Asia and is typically associated with Epstein-Barr virus (EBV) infection. Reactive oxygen species (ROS), as highly reactive oxygen metabolites, play a key role in regulating cellular signaling and maintaining redox homeostasis. Although the role of ROS in tumors has been well established, reviews examining how ROS influence NPC and their potential therapeutic applications remain relatively scarce. ROS exert diverse effects in NPC, including roles in EBV-associated processes, regulation of programmed cell death, metabolic reprogramming, formation of an immunosuppressive tumor microenvironment, and epigenetic remodeling. In addition, natural small-molecule compounds that regulate ROS levels and emerging combination therapy strategies have considerable therapeutic potential in NPC. This review discusses the dual role of ROS in NPC, examines their contributions to disease development, and further explores how these mechanisms could inform the design of ROS-targeted therapies, as well as the associated translational challenges.

## 1 Introduction

Nasopharyngeal carcinoma (NPC) is a type of head and neck malignancy that originates from the mucosal epithelium of the nasopharynx. It is characterized by distinct geographical clustering and etiological heterogeneity. Statistics show that over 75% of NPC cases are concentrated in Southeast Asia and southern China (Sung et al., 2021; Chen et al., 2019). The development of NPC is typically driven by a combination of factors, including host genetic susceptibility, environmental exposures, and viral infections. Among these, Epstein-Barr virus (EBV) infection is highly associated with the development of NPC (Wang HY. et al., 2016; Pathmanathan et al., 1995; Tsao et al., 2017).

Owing to the deep anatomical location of the nasopharynx and its proximity to critical structures, surgery has a limited role in the treatment of NPC. Depending on the tumor stage, patients receive personalized, comprehensive treatment regimens such as radiotherapy, chemotherapy, targeted therapy, or immunotherapy (Feng et al., 2021). Patients with advanced NPC often present with lymph node metastasis and distant metastasis (Chen et al., 2019). Some patients also develop treatment resistance during radiotherapy or chemotherapy, thereby affecting local control rates and long-term survival outcomes. Studies show that the 5-year overall survival rate for patients with advanced NPC is only 67%–77% (Zhang B. et al., 2019). Therefore, the development of new therapeutic approaches and anticancer drugs is of critical importance.

Reactive oxygen species (ROS) are primarily generated through biochemical reactions during cellular respiration (Juan et al., 2021) and exhibit a classic “double-edged sword” effect in tumor initiation, promotion, and progression (Hayes et al., 2020; Niga et al., 2025; Wang et al., 2021a; Liou and Storz, 2010). Low or moderate levels of ROS can function as signaling mediators that support tumor cell survival, proliferation, and adaptation, whereas excessive ROS can induce oxidative damage and cell death (Ramalingam and Rajaram, 2021; Karde et al., 2014). Compared with normal cells, tumor cells exhibit higher levels of oxidative stress, making them more susceptible to ROS-induced damage (Zhang et al., 2015). In NPC, ROS dysregulation is closely linked to several disease-driving processes. EBV-associated signaling, mitochondrial dysfunction, hypoxia, and inflammatory stress can increase ROS production, while sustained oxidative stress can further influence metabolic reprogramming, immune regulation, epigenetic alterations, and cell-fate decisions. Through these interconnected processes, ROS contribute to tumor cell adaptation, malignant progression, and treatment resistance.

NPC cells therefore need to maintain ROS within a dynamic range that is sufficient to support signaling and adaptation but below a lethal threshold. This dynamic balance can be described as a “ROS homeostatic setpoint”, which is maintained through coordinated regulation of ROS production and antioxidant defenses. Under sustained stress, NPC cells remodel these redox-regulatory networks to preserve survival and metabolic adaptation, whereas disruption of this balance may push cells toward oxidative damage and regulated cell death. This context-dependent role of ROS also provides a rationale for therapeutic strategies that target redox homeostasis in NPC.

While the role of ROS in cancer has been extensively studied, comprehensive reviews focusing on ROS dysregulation in NPC, the contribution of ROS to disease progression, and the therapeutic implications of targeting ROS remain limited. Therefore, this review summarizes the major sources and regulatory mechanisms of ROS in NPC, discusses their roles in tumor development and treatment response, and evaluates ROS-targeted therapeutic strategies and the challenges associated with their clinical translation.

## 2 ROS in the NPC

ROS can be categorized into endogenous and exogenous ROS based on the source. They can be generated during normal physiological processes or induced by external factors such as chemicals, radiation, and inflammation. The mitochondrial respiratory chain is the primary source of intracellular ROS, continuously producing ROS during metabolic processes. To maintain redox homeostasis, cells remove excess ROS through antioxidant defense mechanisms (Srinivasan et al., 2017).

### 2.1 Biological properties of ROS

ROS are a class of highly reactive oxidizing molecules that can be classified into two forms: free radicals and non-free radicals. They primarily include the superoxide anion radical (O_2_·-), hydroxyl radical (·OH), and hydrogen peroxide (H_2_O_2_) (Signorello et al., 2020). Most ROS contain an unpaired electron in the outer orbitals, endowing them with high chemical reactivity and making them prone to participating in and initiating free-radical chain reactions (Zhang L. et al., 2019).

At the same time, most metabolic processes within cells involve redox reactions. These reactions facilitate electron transfer between highly reactive molecules, making the generation of ROS inevitable under physiological conditions. To maintain redox homeostasis, the body has developed various antioxidant defense systems, including reduced glutathione, superoxide dismutase, and peroxidases, thereby keeping ROS levels within a range that is harmless to cells (Bindoli et al., 2008).

When ROS accumulate abnormally or the antioxidant system’s function is impaired, the balance between oxidation and antioxidation is disrupted, and excessive ROS can induce oxidative stress and trigger a series of pathological processes.

### 2.2 Sources and dysregulation of ROS generation in NPC

Under physiological conditions, ROS are continuously generated during cellular metabolism, primarily from the mitochondrial respiratory chain, the endoplasmic reticulum (ER), and ROS-producing enzymes such as nicotinamide adenine dinucleotide (NADPH) oxidases (NOXs), while antioxidant systems maintain redox homeostasis to support normal cellular signaling. In NPC, mitochondrial dysfunction, metabolic alterations, ER stress, and EBV-associated factors enhance ROS generation through these pathways, thereby increasing the oxidative burden in NPC cells.

#### 2.2.1 Mitochondrial ROS

Mitochondria are key sites for intracellular redox reactions and electron transport, and they are also the primary sites of ROS production (Srinivasan et al., 2017). During catabolism, substances like glucose and fatty acids enter the tricarboxylic acid (TCA) cycle, generating reduced products such as nicotinamide adenine dinucleotide (NADH) and flavin adenine dinucleotide (FADH2). These provide a source of electrons for subsequent electron-transfer processes and are crucial for maintaining the TCA cycle. NADH and FADH2 transfer electrons to the electron transport chain (ETC) on the inner mitochondrial membrane. In oxidative phosphorylation (OXPHOS), oxygen molecules act as the terminal electron acceptors, undergoing reduction to H_2_O_2_ and thereby driving the synthesis of adenosine triphosphate (ATP) (Nolfi-Donegan et al., 2020; Martínez-Reyes and Chandel, 2020). The TCA cycle is coupled with the OXPHOS, generating large amounts of ATP while consuming oxygen, thereby providing energy for cellular activities. Throughout this process, some electrons leak from the electron transport chain and react with oxygen molecules to form ROS.

Mitochondrial complex I and complex III are the major sites of electron leakage (Balaban et al., 2005; Parascandolo and Laukkanen, 2019). Complex I can directly transfer electrons to oxygen molecules to form superoxide anions, while complex III can transfer electrons to ubiquinone to form semiquinone radicals, which react with oxygen molecules to generate superoxide anions. Manganese superoxide dismutase (SOD2) in the mitochondrial matrix or Cu-Zn superoxide dismutase (SOD1) in the intermembrane space catalyzes the conversion of superoxide anions into hydrogen peroxide (Kim and Kim, 2022). Hydrogen peroxide can not only be further reduced to water by mitochondrial glutathione peroxidase (GPX) or catalase (CAT), but can also react with iron ions to generate highly reactive hydroxyl radicals (Vercellino and Sazanov, 2022). Superoxide dismutase 3 (SOD3) catalyzes the conversion of O_2_·- to H_2_O_2_ in the extracellular space (Fukai and Ushio-Fukai, 2011). Complex II can significantly generate ROS under specific experimental conditions, when complex I and complex III are inhibited and the concentration of succinate is low.

In NPC, dysfunction of mitochondrial respiration, membrane potential, membrane permeability, or energy metabolism results in enhanced electron leakage in the aforementioned physiological processes, consequently increasing the levels of ROS. Recent studies have demonstrated that several mitochondria-associated factors, including p38 mitogen-activated protein kinase γ (p38γ), YME1-like protein 1 (YME1L), and translocase of outer mitochondrial membrane 40 (TOM40), can induce mitochondrial dysfunction and promote ROS accumulation (Cheng et al., 2024; Ran et al., 2023; Yin et al., 2022). Song et al. revealed that the EBV-encoded protein BHRF1, located in mitochondria, can alter mitochondrial membrane permeability, thereby increasing ROS (Song et al., 2020). Furthermore, the level of mitochondrial OXPHOS can also influence the generation of mitochondrial reactive oxygen species (mtROS) (Huang et al., 2024; Chung et al., 2020).

#### 2.2.2 ROS from other sources

In addition to mitochondria, enzymatic pathways are also major sources of ROS in cellular oxidative stress. For example, NADPH oxidase (NOX) (Bevere et al., 2022), nitric oxide synthase (NOS) (Elahi et al., 2009), cytochrome P450 (Kwiatkowska et al., 2021), xanthine oxidase (XO) (Pagan et al., 2022), myeloperoxidase (MPO), and cyclooxygenase (COX) (Lüthje and Martinez-Cortes, 2018) can all contribute to endogenous ROS production. In NPC, dysregulation of these pathways can further increase ROS generation and contribute to oxidative stress.

The NOX family is a major intrinsic source of ROS. Based on enzymatic activity, members of the NOX family are divided into two groups: catalytic enzymes (NOX1-5 and DUOX1-2) and regulatory subunits (p22phox, p40phox, p47phox, p67phox, Rac1, and Rac2) (Lambeth et al., 2007). As important membrane-bound enzymes, NOXs generate superoxide by transferring electrons from the cytoplasm to the extracellular space. In NPC, the inducible activity of membrane NADPH oxidases promotes ROS. Recent studies have shown that EBV-associated proteins LMP1 and EBNA1 can both promote ROS generation by increasing NOX expression (Cao et al., 2012; Sun et al., 2015), while Ras-related C3 botulinum toxin substrate 2 (Rac2) drives hypoxia-related radioresistance in the NPC by enhancing ROS (Chen H. et al., 2025).

The ER is also one of the main sources of ROS. The accumulation of misfolded proteins triggers ER stress, thereby activating unfolded protein response (UPR) to restore protein homeostasis (Marciniak et al., 2022). During the UPR, the coordinated action of protein disulfide isomerase, ER oxidoreductin 1, and NOX complex triggers the ROS cascade (Zeeshan et al., 2016). ER stress releases large amounts of Ca^2+^, increasing the concentration of Ca^2+^ in mitochondria, which in turn affects mitochondrial ROS (Senft and Ronai, 2015). In the context of NPC, upregulation of ER stress-associated proteins triggers programmed cell death in cancer cells via increasing ROS levels (Lin et al., 2016). Furthermore, exogenous stimuli such as ionizing radiation, viral infections, drugs, chemotherapy, and radiotherapy also promote ROS response (Chen et al., 2021; Iic and Da, 2017; Zhuang et al., 2023) (Figure 1).

**FIGURE 1:**
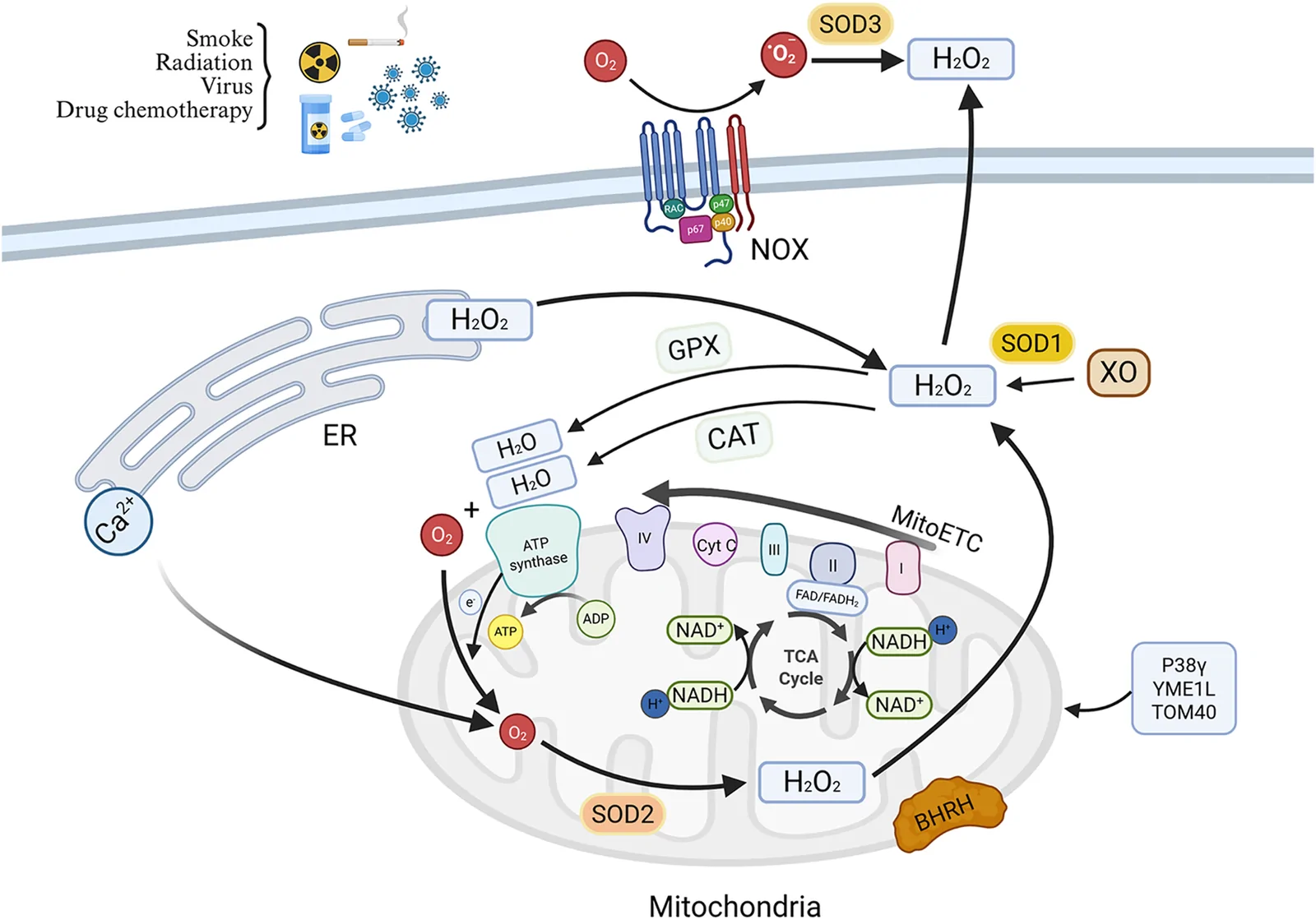
The generation mechanism of ROS. ROS are produced from multiple sources, including mitochondrial electron transport chain (ETC), NADPH oxidases (NOX), xanthine oxidase (XO), and endoplasmic reticulum (ER) stress. Electron leakage from mitochondrial complexes I and III generates superoxide (O_2_·^-^), which is converted into H_2_O_2_ by SODs and further detoxified by GPX and CAT. Mitochondrial dysfunction and EBV-related factors enhance ROS production. Exogenous stimuli such as radiation, viral infection, and chemotherapy also contribute to ROS accumulation, ultimately disrupting redox homeostasis in NPC (Created by the BioRender.com).

### 2.3 Mechanisms for maintaining redox homeostasis in NPC

ROS play a crucial role in regulating normal cellular processes, while dysregulated ROS homeostasis may contribute to pathological states in humans. Some studies show that activate pro-tumorigenic signaling, induce functional impairment in various tissues, and cause structural cellular damage, thereby promoting the onset and progression of cancer (Arfin et al., 2021; Kuo et al., 2022). Non-enzymatic antioxidants, enzymatic antioxidants, and antioxidant genes constitute an integrated antioxidant defense system that safeguards ROS homeostasis (Franco et al., 2019; Gulcin, 2020).

Non-enzymatic antioxidants are capable of directly capturing ROS, controlling the functions of signaling pathways and transcription factors, which ultimately influence the cellular response to stress and adaptation. A typical example of non-enzymatic antioxidants is reduced glutathione (GSH) (Irato and Santovito, 2021). Yang et al. demonstrated that ionizing radiation (IR) induces cell death in NPC cells by decreasing intracellular GSH, increasing ROS, and forming oxidized glutathione (GSSG) (Yang et al., 2024).

Examples of enzymatic antioxidants include SOD and peroxiredoxin (PRDX) (Dietz et al., 2016; Jaiswal et al., 2021). In NPC, different members of the PRDX family exert distinct effects on ROS homeostasis. PRDX1 is primarily induced under high ROS signaling conditions and participates in the adaptive regulation of tumor cells to oxidative stress environments. Mitochondrial peroxiredoxin 3 (PRDX3) plays a crucial role in mitochondrial function and quality control. PRDX3 increases ROS levels, leading to mitochondrial dysfunction and substantially inhibiting the malignant behavior of NPC cells (Qiang et al., 2025). Highly expressed SOD1 maintains intracellular ROS at tolerable levels by limiting the accumulation of superoxide anions, thus supporting tumor cell survival and growth. Conversely, inhibition of SOD1 leads to elevated ROS levels, triggering apoptosis and inhibiting tumor growth (Li S. et al., 2018).

Antioxidant genes are genes that encode non-enzymatic or enzymatic antioxidants, or genes that participate in modulating the expression and function of antioxidants, such as Nrf2 (Wu et al., 2019) and p53 (Achanta and Huang, 2004; Hassin and Oren, 2023). The Nrf2 signaling pathway participates in regulating the radiotherapy response in NPC by modulating intracellular ROS levels. Radiotherapy induces Nrf2 accumulation and nuclear translocation, accompanied by activation of downstream antioxidant responses. Inhibition of Nrf2 leads to ROS accumulation and consequently enhances cellular radiosensitivity. Conversely, knockdown of Kelch-like ECH-associated protein 1 (KEAP1), a negative regulator of Nrf2, enhances Nrf2 signaling, thus promoting ROS clearance and conferring radioresistance (Zhou et al., 2020). Further studies have shown that laminin subunit beta 3 (LAMB3) promotes radioresistance by suppressing forkhead box O3 (FOXO3) expression and activating the Nrf2/HO-1 axis, thereby reducing intracellular ROS levels (Zhuang et al., 2023). In contrast, Ras association domain family member 1 isoform A (RASSF1A) enhances radiosensitivity by promoting FOXO3 expression and inhibiting the Nrf2/thioredoxin reductase 1 (TXNRD1) axis, which results in increased ROS levels (Si et al., 2023). High expression of the p53 downstream target gene TP53-induced glycolysis and apoptosis regulator (TIGAR) promotes the pentose phosphate pathway and increases nicotinamide adenine dinucleotide phosphate (NADPH), leading to enhanced intracellular ROS scavenging and inhibition of oxidative stress-induced apoptosis (Ey et al., 2015).

In addition to classical antioxidant systems, NPC cells can also activate factors related to mitochondrial quality control through epigenetic regulation to reduce ROS levels and prevent the shift of cellular fate toward senescence. Studies have shown that tripartite motif-containing protein 29 (TRIM29) interacts with voltage-dependent anion-selective channel 1 (VDAC1) to activate mitophagy and clear damage mitochondria, thereby reducing ROS levels and inhibiting cellular senescence (Yue et al., 2023). This mechanism is highly consistent with the PRDX3-PINK1/Parkin pathway in function, which further supports the notion that NPC cells regulate ROS at its source by enhancing mitochondrial quality control (Qiang et al., 2025).

## 3 Viral and non-viral drivers of ROS dysregulation in NPC

### 3.1 The reciprocal regulation between ROS and EBV infection

EBV infection is a central pathogenic mechanism underlying the development and progression of NPC. It is characterized by the persistent expression of various viral latent proteins in tumor cells, which consequently reshape the host cell’s metabolic state and signaling networks. Extensive research indicates that ROS serve as a critical molecular link between EBV infection and the biological behavior of NPC.

EBV can regulate ROS production by directly targeting mitochondria. BHRF1, an EBV-encoded protein homolog to Bcl-2, is highly expressed in EBV-infected NPC and is primarily localized to mitochondria. BHRF1 expression induces an increase in cyclophilin D dependent mitochondrial membrane permeabilization transition (MMPT). MMPT further disrupts mitochondrial function, increases ROS production, and activates mitophagy, ultimately enhancing the tumorigenic and metastatic potential of NPC (Song et al., 2020). This study indicates that EBV promotes the development and progression of NPC at the mitochondrial level through the BHRF1-MMPT-ROS-mitophagy axis.

EBV can also increase intracellular ROS by regulating the NADPH oxidase system. EBNA1, a viral nuclear protein expressed in NPC can upregulate several key molecules associated with the oxidative stress response, including SOD1 and Prx1. Prolonged high expression of EBNA1 elevates NOX1 and NOX2 levels, leading to increased ROS. Furthermore, EBNA1 depletion in EBV-positive NPC cells reduces NOX2 and ROS. These findings suggest that EBNA1 regulates the oxidative stress response via the NOX1/2-ROS axis, thereby promoting tumorigenesis (Cao et al., 2012).

EBV latent membrane protein 1 (LMP1) also plays a crucial role in ROS regulation. LMP1 can activate the JNK pathway to upregulate the expression of the NADPH oxidase regulatory subunit p22phox, thus activating NADPH oxidase and inducing excessive ROS (Sun et al., 2015). LMP1-mediated ROS accumulation can further enhance EBV reactivation. EBV disrupts the “ROS homeostatic setpoint” by upregulating the expression of NOX2 and Nrf2, leading to ROS accumulation and a state of high oxidative stress. This state of elevated oxidative stress is associated with EBV reactivation and may contribute to radioresistance and an increased risk of recurrence. Additionally, 8-hydroxy-2′-deoxyguanosine (8-OHdG), a biomarker of oxidative DNA damage, combined with EBV DNA can serve as a dual prognostic marker for identifying high-risk patients with poor outcomes (Hu et al., 2020). This implies a dual mechanism in which NOX-mediated ROS accumulation and metabolic rewiring operate in concert to sustain the malignant phenotype of EBV-positive NPC (Sun et al., 2015).

ROS-mediated inflammasome activation is a critical link connecting oxidative stress and tumor immune regulation in EBV-associated NPC. Multiple inflammasome pathways are activated in EBV-associated NPC. Absent in melanoma 2 (AIM2) and retinoic acid-inducible gene I (RIG-I) are involved in IL-1β production induced by EBV genomic DNA and EBV-encoded small RNAs, respectively, while the NOD-like receptor family pyrin domain-containing 3 (NLRP3) responds to extracellular ATP and ROS. In mouse models, tumor-derived IL-1β suppresses tumor growth by recruiting immunostimulatory neutrophils. Moreover, elevated expression of inflammasome components in NPC tumors is associated with improved patient survival (Chen et al., 2012).

### 3.2 Potential mechanisms of ROS dysregulation in EBV-negative NPC

Although EBV is a major viral factor associated with NPC, EBV-negative NPC shows considerable virological heterogeneity, including HPV-associated EBV-negative and EBV/HPV double-negative NPC (Kano et al., 2017; Svajdler et al., 2016). North American studies further suggest a nearly mutually exclusive distribution of HPV-positive and EBV-positive NPC (Huang et al., 2022). However, EBV negativity does not exclude oxidative stress, as ROS dysregulation may also arise from other viral factors, tumor-intrinsic alterations, and non-viral stressors.

In NPC without an identified viral driver, tumor metabolic state and microenvironmental stress may contribute to ROS regulation. EBV/HPV double-negative NPC constitutes a proportion of cases in some non-endemic cohorts and is associated with unfavorable outcomes (Stenmark et al., 2014). Although direct studies of redox homeostasis in this subtype remain limited, virus-negative NPC cell models indicate that ROS regulation can occur without a defined viral driver. In HK1 cells, in which no known viral infection was detected (Strong et al., 2014), 3-hydroxy-3-methylglutaryl-CoA lyase (HMGCL), a key enzyme involved in ketogenesis, regulates intracellular ROS through ketone-body metabolism (Luo et al., 2017). Nutrient deprivation also activates ROS-dependent adaptive signaling that supports tumor cell survival (Wang CC. et al., 2025), while EBV-negative NPC cells can accumulate lipid ROS under oxidative stress (Yuan et al., 2022). Together, these findings indicate that EBV is not the sole determinant of redox dysregulation in NPC; metabolic alterations, nutrient stress, and lipid oxidation may also shape the redox state of EBV-negative tumors.

Beyond these non-viral mechanisms, high-risk HPV may represent an alternative viral background in a subset of EBV-negative NPC. HPV16 and HPV18 are among the more common genotypes in HPV-positive NPC (Zhao et al., 2024). In HPV-related head and neck cancers, the viral oncoproteins E6 and E7 inactivate p53 and retinoblastoma protein (pRb), respectively. However, the mechanisms by which HPV contributes to NPC remain poorly defined (Kano et al., 2017). Other HPV-associated tumor models indicate that HPV proteins can alter cellular redox homeostasis through E6/E7-mediated NOX2-dependent ROS generation (Marullo et al., 2015), E6-associated mitochondrial metabolic changes (Cruz-Gregorio et al., 2019), and differential regulation of antioxidant defenses by viral early proteins (Cruz-Gregorio et al., 2018). These findings provide a potential mechanistic basis for redox abnormalities in HPV-associated EBV-negative NPC. However, most mechanistic evidence comes from HPV-related head and neck tumors or other epithelial models. The relevance to HPV-positive NPC therefore requires direct validation.

## 4 The central role of ROS in the development and progression of NPC

The biological effects of ROS in NPC are highly dependent on both dose and cellular context. Rather than being determined by ROS levels alone, these effects reflect the balance between ROS production and antioxidant defenses (Cheung and Vousden, 2022; Glorieux et al., 2024). At basal or sublethal levels, ROS act as signaling intermediates that support cell proliferation, survival, and metabolic regulation, whereas sustained but manageable oxidative stress can further promote metabolic rewiring, immune modulation, and epigenetic adaptation (Wang et al., 2024; Qian et al., 2025). Once ROS accumulation overwhelms the cellular antioxidant capacity, however, oxidative damage can redirect cell fate from stress adaptation toward autophagy or regulated cell death, including apoptosis, ferroptosis, pyroptosis, and necroptosis (Qian et al., 2025). Thus, ROS function across a dynamic redox spectrum in NPC, ranging from adaptive signaling to cytotoxic stress, providing a conceptual basis for subsequent ROS-targeted therapeutic strategies.

### 4.1 ROS within the adaptive range: redox signaling and tumor adaptation

#### 4.1.1 ROS and metabolic reprogramming

Metabolic reprogramming supports the growth and proliferation of tumor cells in the nutrient-deprived tumor microenvironment (TME) (Chen and Peng, 2026). In NPC, there is a complex interplay between ROS and metabolic reprogramming. On the one hand, ROS can drive alterations in tumor cell metabolic pathways. On the other hand, the remodeling of metabolic pathways can regulate intracellular redox homeostasis, collectively promoting tumor cell growth and malignant progression.

Oncogenic signals can promote metabolic alterations by regulating ROS production. Studies have shown that LMP1 activates NOX to stimulate excessive ROS production and significantly enhances anaerobic glycolytic activity via the PI3K/Akt signaling pathway, thereby promoting metabolic reprogramming in tumor cells (Sun et al., 2015). Conversely, tumor cells can regulate ROS levels through metabolic pathway alterations to adapt to a stressful environment. TIGAR inhibits glycolysis and promotes glucose entry into the pentose phosphate pathway, thereby increasing NADPH production and the capacity to scavenge ROS. TIGAR reduces ROS levels and protects cells from ROS-induced apoptosis, thereby promoting cell survival under oxidative stress (Ey et al., 2015). Malic enzyme 1 (ME1) is an important source of cytosolic NADPH and contributes to the maintenance of intracellular redox homeostasis. Studies have shown that NADPH generated by ME1 not only participates in fatty acid synthesis but also plays a key role in maintaining intracellular redox status and protecting cells from oxidative stress. Inhibition of ME1 reduces the tolerance of NPC cells to low-glucose environments and impairs their migration and invasion capabilities (Zheng et al., 2012).

ROS can also participate in the regulation of energy metabolism in tumor cells by affecting mitochondrial function. In tumor cells, the phenomenon of increased glucose consumption and reliance on aerobic glycolysis for ATP production is known as the Warburg effect. When the glycolytic pathway is disrupted, changes in ROS levels are often accompanied by mitochondrial dysfunction. Studies have shown that the glycolysis inhibitor 3-bromopyruvate (3-BrPA) can inhibit ATP production in tumor cells while inducing massive ROS generation and reducing the mitochondrial membrane potential, thereby promoting cell death (Zou et al., 2015). Lactate dehydrogenase (LDHA) catalyzes the conversion of pyruvate to lactate and plays a key role in the glycolytic process. Zhai et al. demonstrated that LDHA is overexpressed in NPC and is associated with poor prognosis. LDHA inhibitors can significantly increase mtROS production and promote tumor cell apoptosis, consequently enhancing sensitivity to radiotherapy (Zhai et al., 2013). Additionally, certain drugs can alter ROS levels by interfering with the Warburg effect. For example, sevoflurane suppresses NPC cell proliferation and migration by decreasing glucose consumption and lactate production, elevating ROS levels, and disrupting mitochondrial membrane potential (Peng and Zhou, 2024).

In NPC cancer stem cells (CSCs), ROS are also closely associated with metabolic reprogramming. Studies have shown that NPC CSCs undergo metabolic reprogramming, characterized by enhanced glycolytic dependence and concurrent mitochondrial remodeling. NPC CSCs also exhibit low ROS levels and a high mitochondrial membrane potential (Δψm). Enhanced antioxidant defense mechanisms confer a survival advantage on CSCs by reducing ROS levels, thereby increasing their tolerance to radiotherapy and chemotherapy. When the glycolytic pathway is blocked or ROS levels are modulated through metabolic intervention, the survival advantage of CSCs is significantly compromised (Shen et al., 2015). Furthermore, resveratrol suppresses CSC stemness through increased intracellular ROS and inhibition of metabolic switching, with reduced self-renewal, tumor-initiating capacity, and metastatic potential (Shen et al., 2013). These studies suggest that targeting the ROS-metabolic axis may offer a new potential strategy for NPC treatment.

#### 4.1.2 ROS shape the immunosuppressive microenvironment

In NPC, ROS not only regulate tumor cell metabolism and fate but also function as intercellular stress signals that mediate crosstalk between tumor cells and immune cells, thereby reshaping the tumor immune microenvironment.

One important aspect of this regulation involves changes in the composition and function of immune cell populations. Patients with primary NPC exhibit a significantly increased proportion of immunosuppressive CD19^+^CD138^−^IgA^+^B cells, which decreases markedly after radiotherapy. This reduction has been associated with radiotherapy-induced ROS release in the tumor microenvironment, which may selectively eliminate this cell population. The decrease in CD19^+^CD138^−^IgA^+^B cells is accompanied by an increase in cytotoxic T lymphocytes (CTLs), thereby enhancing antitumor immunity (Li et al., 2019a). In EBV DNA-positive NPC patients with concurrent chronic hepatitis B (CHB), lectin-like oxidized low-density lipoprotein receptor-1 (LOX-1)-positive polymorphonuclear myeloid-derived suppressor cells (PMN-MDSCs) increase ROS through endoplasmic reticulum stress and ROS-related pathways, leading to the inhibition of T-cell activation. This promotes an immunotolerant state in these patients and increases the risk of recurrence (Li et al., 2021).

Conversely, reduced ROS production can also contribute to immune suppression and therapeutic resistance in specific contexts. The E3 ubiquitin ligase PJA1 reduces mitochondrial ROS (mtROS) production by promoting the degradation of the mitochondrial phosphatase PGAM5, thereby suppressing CD8^+^ T cell-mediated antitumor immunity and contributing to chemotherapy resistance (Huang et al., 2024). ROS can also act as danger signals involved in inflammasome activation, with treatment-induced IL-1β release contributing to antitumor responses in NPC (Chen et al., 2012).

#### 4.1.3 ROS and epigenetic modifications

Epigenetic modifications regulate gene expression without altering the DNA sequence and include mechanisms such as histone modifications, DNA methylation, and non-coding RNA-mediated regulation (Steyn et al., 2019). Because multiple epigenetic regulators are sensitive to oxidative stress, redox homeostasis and epigenetic regulation are closely interconnected. In NPC, emerging evidence indicates that epigenetic alterations can reshape cellular redox states and thereby influence tumor progression and therapeutic response.

Histone-modifying enzymes can influence redox homeostasis and treatment response in NPC. The N-terminal tails of histones undergo post-translational modifications, including acetylation and methylation, which alter chromatin accessibility and transcriptional regulation. CYLD, a deubiquitinase with tumor-suppressive functions, binds to class I histone deacetylases HDAC1 and HDAC2 through its N-terminal domain and inhibits HDAC1 activity. Transcriptomic analysis further showed that the CYLD-HDAC axis suppresses Nrf2 and its downstream antioxidant genes, resulting in ROS accumulation, oxidative stress, and DNA damage and ultimately enhancing NPC radiosensitivity (Li Y. et al., 2024). In addition, combined treatment with the proteasome inhibitor bortezomib and class I HDAC inhibitors induces ROS-dependent endoplasmic reticulum stress and apoptosis in NPC cells (Hui and Chiang, 2014). In EBV-positive NPC cells, bortezomib combined with suberoylanilide hydroxamic acid (SAHA) enhances ROS-driven apoptosis while suppressing SAHA-induced EBV lytic replication and production of infectious virions (Hui et al., 2013).

DNA methylation induces gene silencing by inhibiting transcriptional activators from accessing target sites or by recruiting meyhyl-binding protein. DNA methylation shapes distinct phenotypes by bidirectionally regulating ROS homeostasis. NOR1 (oxidored-nitro domain-containing protein 1), also known as OSCP1, functions as a tumor suppressor and is frequently downregulated in NPC, with reduced expression associated with poor prognosis. Restoration of NOR1 expression suppresses autophagy, disrupts mitochondrial energy metabolism, and enhances apoptosis under oxidative stress, whereas promoter hypermethylation represents a major mechanism underlying NOR1 silencing (Yi et al., 2017). In the context of radiotherapy, OTU deubiquitinase 1 (OTUD1), a deubiquitinating enzyme, enhances radiosensitivity in NPC by promoting ROS accumulation and apoptosis through deubiquitination and stabilization of SLC25A11. In contrast, hypermethylation of transcription factor AP-2 gamma (TFAP2C) in radiotherapy-resistant NPC cells leads to decreased expression of both TFAP2C and OTUD1. Studies have found that combining the methylation inhibitor decitabine (DAC) with radiotherapy can overcome radiation resistance via the TFAP2C-OTUD1-SLC25A11 axis, significantly improving treatment efficacy (Zhang H. et al., 2025). Yue et al. found that TRIM29 overexpression in NPC cells was associated with low methylation. Silencing TRIM29 not only restricts the growth of NPC cells *in vitro* and *in vivo* but also induces cellular senescence, accompanied by increased ROS levels. In clinical samples, low TRIM29 methylation was detectable in nasopharyngeal swab DNA, suggesting that its abnormal methylation status has potential value for early detection (Yue et al., 2023).

Non-coding RNAs provide an additional layer of epigenetic regulation by modulating gene expression at transcriptional and post-transcriptional levels. The long noncoding RNA radiation-resistant but ferroptosis-vulnerable (RRFERV) enhances NPC malignant progression and radioresistance by promoting TEA domain transcription factor 1 (TEAD1) expression through competitive binding to miR-615-5p and miR-1293. The RRFERV-TEAD1 signaling axis can also regulate lipid peroxidation by transcriptionally activating acyl-CoA synthetase long-chain family member 4 (ACSL4)/transferrin receptor (TFRC). Oxidative stress within radioresistant cells maintains a delicate balance, rendering them more sensitive to ferroptosis inducers. This provides a new therapeutic strategy for overcoming NPC radioresistance (Xu et al., 2025). MicroRNAs can likewise regulate radioresistance by modulating ROS-related signaling. miR-17-5p is upregulated in radioresistant NPC and promotes radioresistance through the PTEN/AKT pathway. Its overexpression reduces irradiation-induced ROS accumulation and protects NPC cells from apoptosis, whereas inhibition of AKT reverses these effects (Hu et al., 2019).

### 4.2 ROS approaching and crossing the adaptive threshold: cell-fate switching

In NPC, ROS regulate the selection and switching of cell death pathways depending on the balance between stress intensity and antioxidant capacity. When ROS levels stay within a controllable range, cells mainly maintain homeostasis via autophagy and antioxidant systems. Once ROS accumulation approaches or exceeds the adaptive buffering capacity of NPC cells, redox signaling can shift from tumor-supportive adaptation toward cell-fate disruption. Depending on the cellular context, antioxidant capacity, and molecular execution machinery, excessive ROS may promote autophagy-associated stress responses or regulated cell death, including apoptosis, ferroptosis, pyroptosis, and necroptosis.

#### 4.2.1 Autophagy

Autophagy is a process in which eukaryotic cells use lysosomes to remove damaged organelles and cytoplasmic proteins under the regulation of autophagy related genes (ATGs). Autophagy is characterized by an increased LC3-II to LC3-I ratio, decreased p62 levels, and the formation of autophagic vesicles (Kroemer and Jäättelä, 2005). ROS-driven autophagy plays a bidirectional regulatory role in carcinogenesis and can be classified into protective autophagy and stress-induced autophagy. In models involving isogarcinol, ginsenoside Rg1, geraniin, triptonide, and jervine, autophagy enhancement exerts anticancer effects (Li J. et al., 2024; Chen J. et al., 2020; Li et al., 2019b; Chen et al., 2022; Geng et al., 2025). In contrast, studies on coronarin D show that inhibiting autophagy paradoxically enhances cytotoxicity, suggesting that autophagy can protect tumors under specific conditions (Chen et al., 2017).

In NPC, elevated ROS levels are often accompanied by a decrease in mitochondrial membrane potential and functional imbalance. These changes collectively contribute to the early signaling of autophagy initiation. Isogarcinol induces mitochondrial dysfunction and ROS overproduction by reducing the mitochondrial membrane potential. Simultaneously, isogarcinol promotes the conversion of LC3-I to LC3-II, but increases p62 levels to block autophagic flux. This results in the accumulation of damaged mitochondria, which further elevates ROS and drives autophagy-associated stress, ultimately exerting antitumor activity (Li J. et al., 2024). Ni et al. demonstrated that Parkin overexpression can elevate ROS and LC3-I/LC3-II by activating the BCL2-interacting protein 3 (BNIP3)/NIX signaling, thereby promoting mitochondrial autophagy to enhance sensitivity to paclitaxel in NPC (Ni et al., 2023).

ROS can also regulate autophagy by modulating energy metabolism and stress pathways. Cyclin B1 knockdown elevates ROS, thereby activating autophagy through ROS-mediated AMPK/ULK1 signaling (Xie et al., 2019). Jervine induces ROS accumulation in NPC cells, concomitantly blocking AKT/mTOR signaling while activating AMPK, which collectively drives autophagy (Chen J. et al., 2020). Furthermore, studies show that ginsenoside Rg1 and geraniin trigger autophagic cell death by inhibiting the PI3K/AKT/mTOR signaling pathway and elevating ROS and LC3 conversion (Li et al., 2019b; Chen et al., 2022). *In vitro* experiments reveal that triptonide induces autophagy in NPC cells by activating p38 MAPK signaling and inhibiting mTOR/NF-κB pathways in the context of elevated ROS (Geng et al., 2025). Additionally, coronarin D promotes autophagy through ROS elevation and JNK pathway activation (Chen et al., 2017) (Figure 2).

**FIGURE 2:**
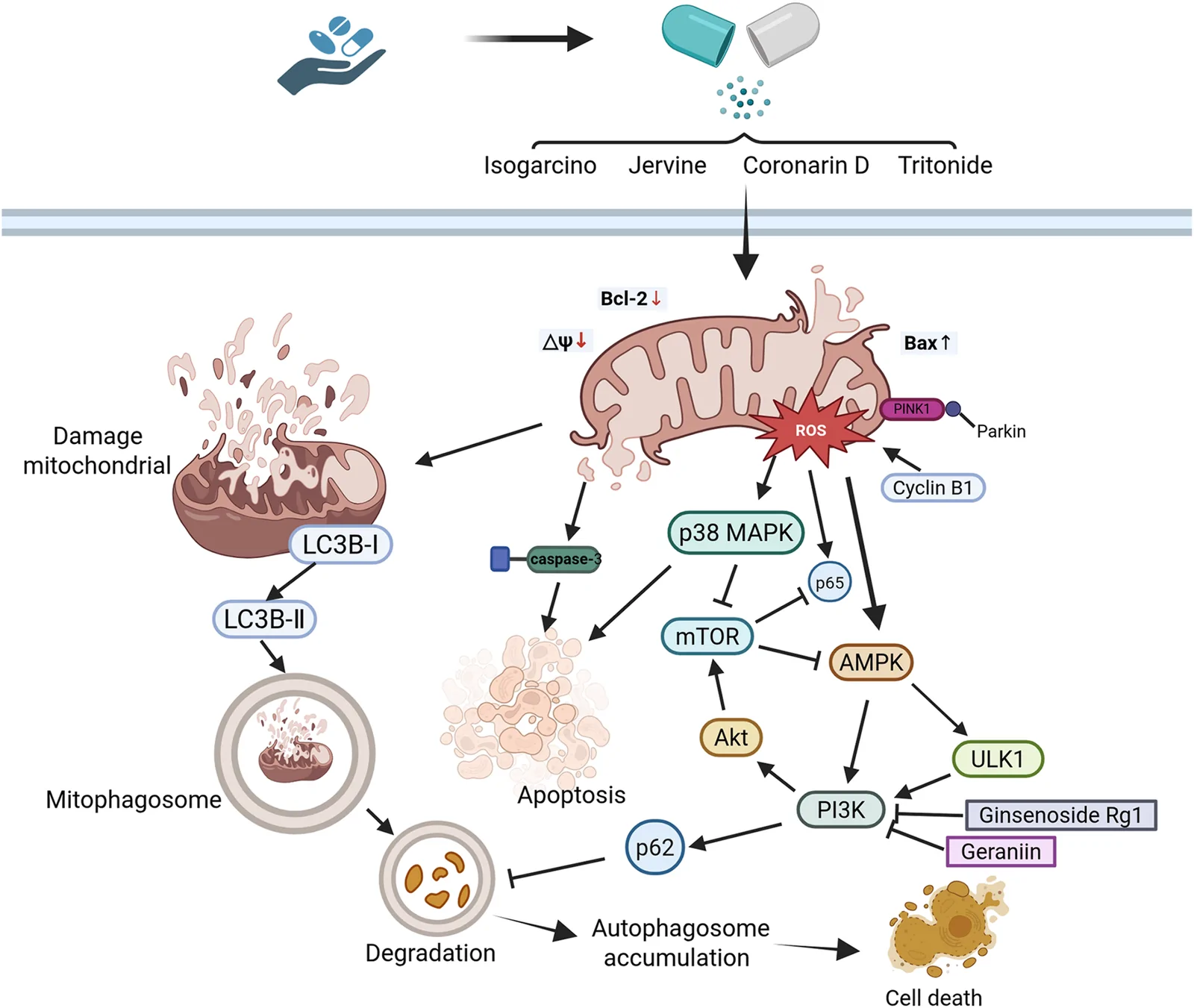
The mechanism of ROS-mediated autophagy in NPC. ROS accumulation induces mitochondrial dysfunction, including decreased membrane potential (Δψ) and altered Bcl-2/Bax balance, thereby promoting mitophagy and apoptosis. ROS regulates autophagy through multiple signaling pathways, including AMPK-ULK1, p38 MAPK, and PI3K/Akt/mTOR. These pathways contribute to LC3 conversion, p62 regulation, and autophagosome formation. Mitochondrial damage and ROS-related signaling further enhance autophagic activity. Exogenous compounds such as isogarcinol, jervine, ginsenoside Rg1, geraniin, triptonide, and coronarin D also modulate ROS, thereby influencing autophagic flux and cell fate in NPC. (Created by the BioRender.com).

#### 4.2.2 Apoptosis

Apoptosis is an active, programmed cell death process that occurs through caspase family cascade reactions. Apoptosis can be executed via the mitochondria-dependent intrinsic pathway or the death-receptor-mediated extrinsic pathway.

In NPC cells, excessive ROS accumulation serves as a key upstream signal triggering apoptosis. ROS can induce apoptosis via the mitochondrial apoptosis pathway. The absence of the DNA damage response-associated factors NFBD1/MDC1 leads to a p53-dependent increase in intracellular ROS levels, which in turn activates the mitochondrial apoptosis pathway (Wang Z. et al., 2017). Multiple drug-treatment models have shown that elevated ROS levels are typically accompanied by changes in mitochondrial membrane potential, cytochrome c release, and caspase-9/3 activation, as well as downregulation of the anti-apoptotic protein Bcl-2 and upregulation of the pro-apoptotic protein Bad. Elevated ROS can also mediate apoptosis by reducing phosphorylated AKT (p-AKT) levels via the PI3K/AKT pathway (Yan et al., 2011; Lin et al., 2015).

In addition to acting directly on mitochondria, ROS can also participate in and amplify apoptosis dose- and time- dependent by inducing ER stress. Elevated intracellular ROS levels typically occur in the early stages and precede the onset of ER stress and mitochondrial dysfunction. As ROS levels rise, ER stress markers are induced in a dose-and time-dependent manner, further leading to loss of mitochondrial membrane potential, cytochrome c release, and the activation of caspases 4, 9, and 3. These findings indicate that ROS act as upstream signals triggering the ER stress-AKT inactivation-mitochondrial apoptosis axis (Liu et al., 2011).

Notably, ROS can also serve as a key signal linking extrinsic and intrinsic apoptotic pathways. AG4, a saponin component isolated from Rhizoma Ardisiae Gigantifoliae, has shown antitumor activity in NPC cells. AG4-induced apoptosis is accompanied by a rapid and sustained increase in ROS. This process simultaneously activates the Fas/FasL-mediated death receptor pathway and the Bcl-2 family-regulated mitochondrial signaling pathway. This suggests that ROS may act as a key factor in multiple apoptotic signaling pathways, collectively determining the overall activation status of the apoptotic program (Dong et al., 2015) (Figure 3).

**FIGURE 3:**
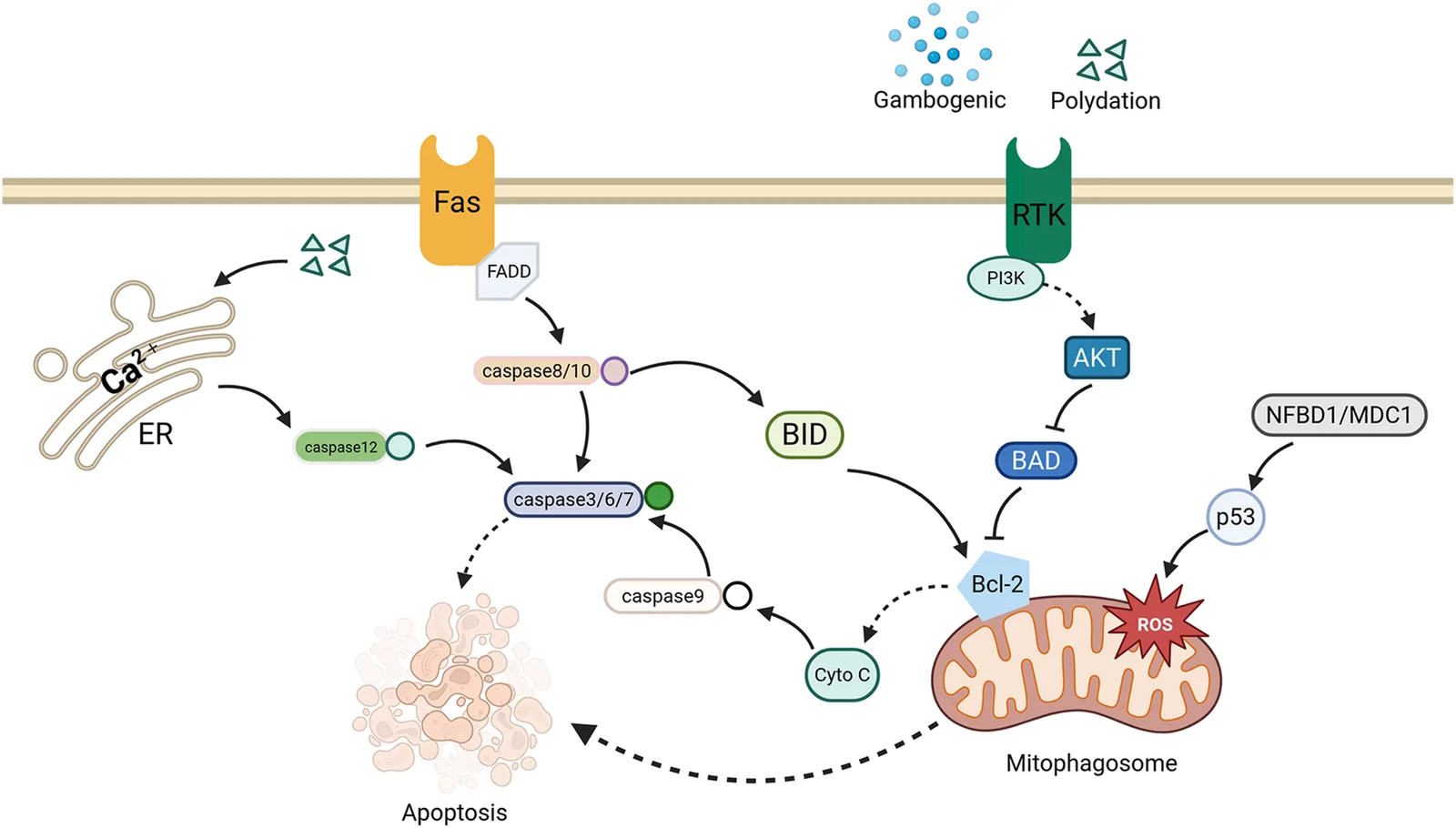
The mechanism of ROS-mediated apoptosis in NPC. ROS accumulation induces apoptosis via mitochondrial (intrinsic) and death receptor-mediated (extrinsic) pathways. It promotes mitochondrial dysfunction, cytochrome c release, caspase activation, and Bcl-2 family regulation, and modulates apoptosis through PI3K/Akt inhibition and p53 activation. ROS also triggers ER stress, leading to caspase-12 activation and amplification of mitochondrial apoptosis, while linking extrinsic and intrinsic pathways via Fas/FasL signaling and BID. Exogenous compounds further enhance ROS accumulation and apoptotic cell death in NPC (Created by the BioRender.com).

#### 4.2.3 Ferroptosis

Ferroptosis is an iron-dependent form of regulated cell death driven by the accumulation of lipid peroxides in cellular membranes, ultimately resulting in irreversible membrane damage and cell death. Ferroptosis is characterized by mitochondrial shrinkage and cristae reduction, which significantly distinguishes it from caspase-dependent apoptotic body formation (Lei et al., 2022). ROS levels plays a key role in regulating lipid peroxidation and ferroptosis. When ROS production is persistently elevated and the capacity to clear lipid peroxides is limited, the cell death pathway can shift from apoptosis-characterized by mitochondrial damage and caspase activation-to ferroptosis, which is centered on uncontrolled iron-dependent lipid peroxidation.

The molecular basis for this pathway switch lies in alterations in the regulatory capacity of the System Xc^−^(SLC7A11/SLC3A2)-GSH-GPX4 antioxidant axis to regulate ROS and lipid peroxidation (Wu et al., 2024). Impairment of this axis leads to decreased glutathione (GSH) synthesis and restricted glutathione peroxidase 4 (GPX4) function, thereby weakening the capacity to clear lipid peroxides. This process is associated with elevated intracellular ROS, lipid peroxidation products (e.g., MDA), and Fe^2+^ levels, alongside the manifestation of ferroptosis-related phenotypes, including mitochondrial shrinkage, cristae reduction, and increased mitochondrial membrane density (Wang et al., 2024; Wang J. et al., 2025). Similar studies show that inhibiting GSH synthesis or reducing GPX4 levels can further increase levels of lipid peroxides and ROS, thereby triggering ferroptosis (Li X. et al., 2024). In radiation therapy-related models, upregulation of thioredoxin-interacting protein (TXNIP) can promote ferroptosis by activating the xCT-GSH-GPX4 axis and enhancing ROS (Zhou et al., 2025).

In addition to the aforementioned core antioxidant execution axis, some studies suggest that ferroptosis may also be accompanied by alterations in the state of stress signaling pathways. In an isoquercitrin-treated NPC model, AMPK/NF-κB p65 pathway inhibition was observed concomitantly with elevated ROS and ferroptosis (Luo et al., 2024). Furthermore, factors in the tumor microenvironment can alter the state of the ROS buffering system through upstream regulatory nodes. Under hypoxic conditions, HIF-1α-mediated BAP1 upregulation suppresses SLC7A11 expression via modulation of histone H2A stability, consequently disrupting System Xc^−^ function. This makes tumor cells more prone to generating lipid ROS to enhance ferroptosis, thereby improving chemotherapy resistance in NPC (Cai et al., 2024) (Figure 4).

**FIGURE 4:**
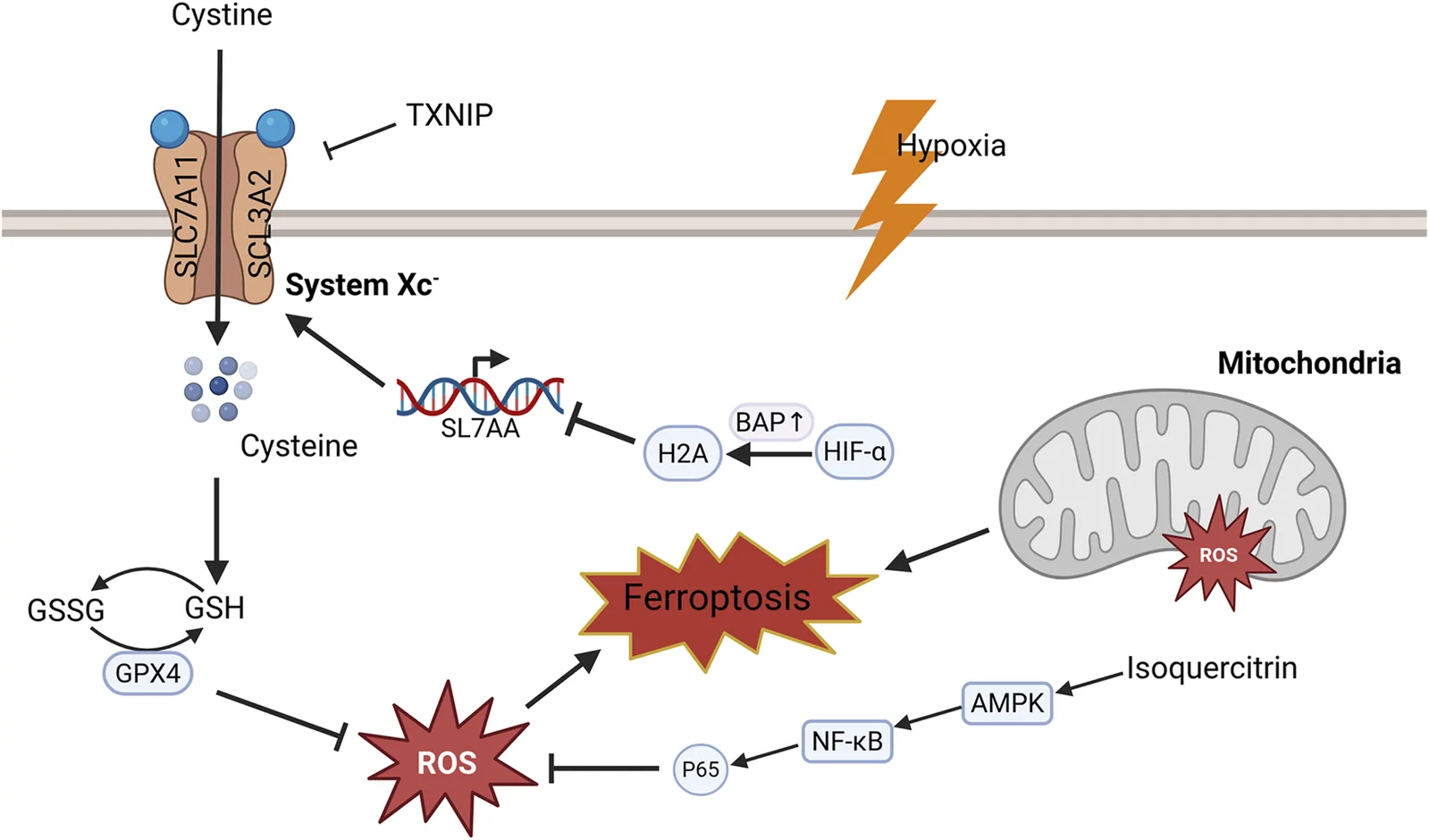
The mechanism of ROS-mediated ferroptosis in NPC. ROS accumulation promotes ferroptosis by inducing lipid peroxidation and disrupting redox homeostasis. This process is mainly regulated by the System Xc^−^-GSH-GPX4 axis, where inhibition of SLC7A11-mediated cystine uptake reduces glutathione (GSH) synthesis and impairs GPX4 activity, leading to lipid peroxide accumulation. Elevated ROS further amplifies lipid peroxidation and ferroptotic cell death. Ferroptosis is also modulated through AMPK/NF-κB signaling. In addition, hypoxia regulates ferroptosis via the HIF-1α/BAP1 axis, suppressing SLC7A11 expression and enhancing ROS accumulation. Exogenous compounds such as isoquercitrin further promote ferroptotic cell death in NPC (Created by the BioRender.com).

#### 4.2.4 Pyroptosis

Pyroptosis is a lytic form of programmed cell death characterized by the formation of membrane pores after the cleavage of Gasdermin family proteins, which leads to cell swelling, rupture, and the release of inflammatory mediators. Gasdermin D (GSDMD), a key pore-forming effector of pyroptosis, is cleaved by activated caspase-1 to release its pore-forming N-terminal fragment, thereby disrupting plasma membrane integrity. Gasdermin E (GSDME), another pore-forming member of the gasdermin family, can be cleaved by caspase-3 to induce pyroptotic membrane permeabilization. In the classical pyroptosis pathway, caspase-1 executes pyroptosis by cleaving GSDMD. However, in some cases, caspase-3-mediated cleavage of GSDME can also induce pore formation and produce a pyroptosis-like phenotype (Wang Y. et al., 2017). Inflammasome activation, caspase cleavage, and pore formation are all highly sensitive to the intracellular redox status, and ROS serve as a key regulatory factor linking stress signals to pyroptosis.

In NPC, the classical inflammasome-caspase-1-GSDMD pathway is associated with enhanced mtROS. Upon activation, the inflammasome recruits apoptosis-associated speak-lile protein containing a CARD (ASC) to form ASC speak, which activate caspase-1 and pro-inflammatory cytokines (e.g., IL-1β), thereby initiating pyroptosis (Hoss et al., 2017; Kovacs and Miao, 2017). Multiple lines of evidence suggest that mtROS can act as an upstream regulator of NLRP3 inflammasome activation (Liu et al., 2018; Chung et al., 2018). Previous studies have demonstrated that in NPC cells treated with cisplatin and forming ASC specks, multiple mitochondrial oxidative phosphorylation (OXPHOS) complex-related proteins, including NDUFB3, NDUFB8, and ATP5, are significantly upregulated, thereby promoting mtROS generation. The elevated mtROS subsequently activates the NLRP3 inflammasome, accompanied by caspase-1 activation and increased IL-1β secretion, ultimately driving pyroptosis. In clinical samples, high-level expression of NDUFB8 and ATP5B was significantly associated with better local recurrence-free survival, suggesting that the OxPhos-mtROS-NLRP3 inflammasome axis influences the prognosis of local tumor recurrence by regulating pyroptosis (Chung et al., 2020). Tanshinone IIA treatment of NPC HK1 cells promotes pyroptosis by regulating the miR-125b-FOXP3-caspase-1-GSDMD signaling axis. During this process, elevated ROS levels and increased LDH release suggest a close association between pyroptosis and oxidative stress (Wang et al., 2021b).

In addition to the caspase-1-GSDMD pathway, NPC can also undergo pyroptosis via the caspase-3-GSDME pathway. Cisplatin induces proteasomal degradation of cellular inhibitor of apoptosis proteins 1 and 2 (cIAP1/2), thereby mediating pyroptosis through the caspase-3-GSDME pathway. The cIAP1/2 antagonist birinapant significantly enhances the caspase-3-GSDME-mediated pyroptosis induced by cisplatin both *in vitro* and *in vivo*. Further studies show that inhibiting ROS levels and caspase-3 cleavage can reverse this pyroptotic effect (Chen Z. et al., 2020). Huang et al. found that the E3 ubiquitin ligase PJA1 suppresses GSDME-mediated pyroptosis by increasing the ubiquitination of the mitochondrial protein PGAM5 and reducing mtROS production, thereby promoting docetaxel resistance. This study suggests that the inhibition of ROS and pyroptosis is associated with chemotherapy resistance (Huang et al., 2024) (Figure 5).

**FIGURE 5:**
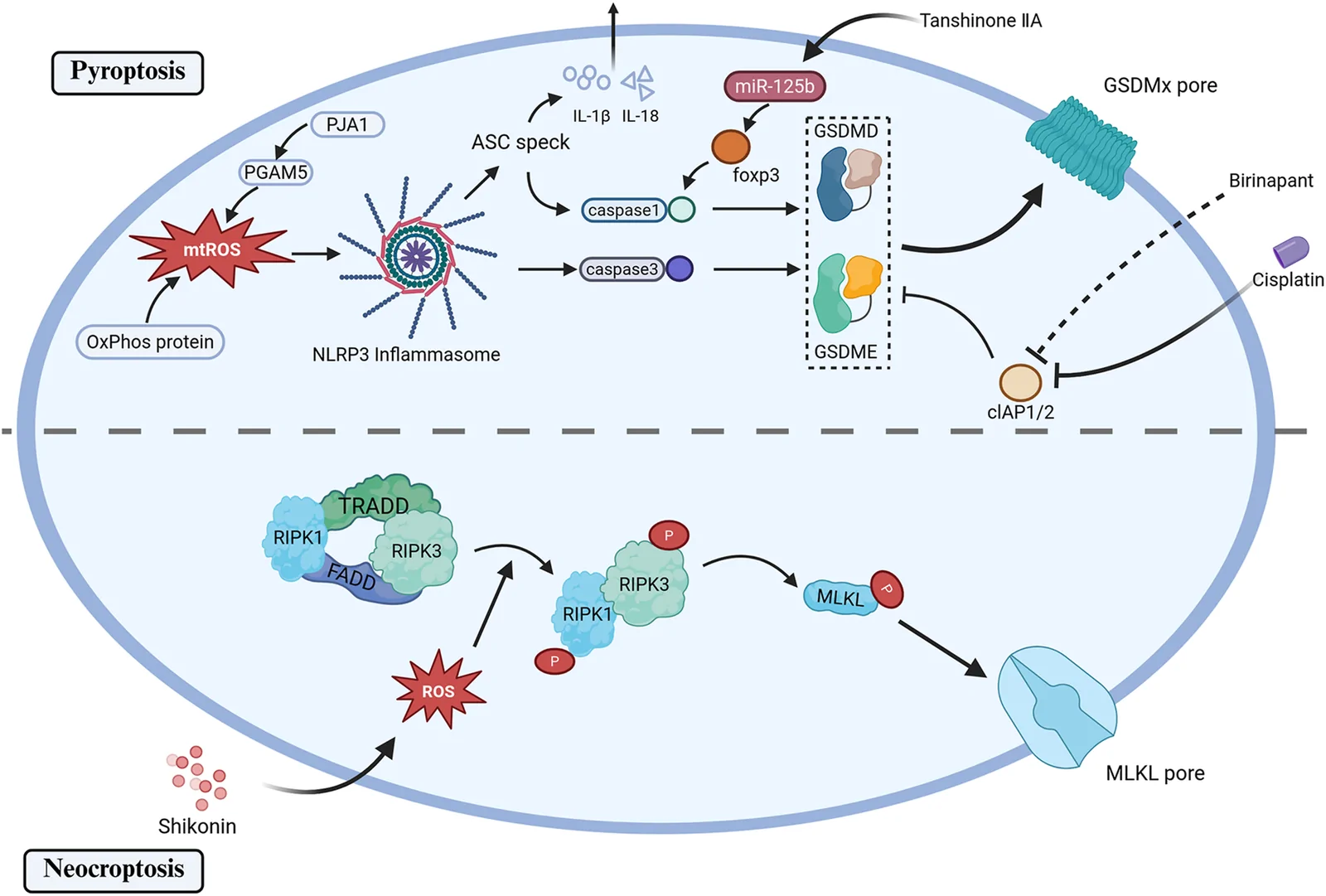
The mechanisms of ROS-mediated pyroptosis and necroptosis in NPC. ROS accumulation induces pyroptosis through inflammasome activation and caspase-dependent pathways. Elevated mtROS promotes NLRP3 inflammasome assembly, ASC speck formation, and caspase-1 activation, leading to GSDMD cleavage, membrane pore formation, and release of pro-inflammatory cytokines (e.g., IL-1β and IL-18). ROS also mediates pyroptosis via the caspase-3-GSDME pathway. In parallel, ROS can trigger necroptosis through activation of the RIPK1/RIPK3/MLKL signaling pathway, resulting in MLKL phosphorylation and membrane pore formation. Although the mechanism of necroptosis in NPC remains less well characterized, it shares structural similarities with pyroptosis, where RIPK1/RIPK3 activation functionally parallels caspase activation, and MLKL-mediated membrane disruption corresponds to gasdermin-dependent pore formation. Exogenous compounds such as cisplatin, tanshinone IIA, birinapant, and shikonin modulate these processes by altering ROS levels, thereby influencing inflammatory cell death in NPC (Created by the BioRender.com).

#### 4.2.5 Necroptosis

Under certain stress conditions where canonical caspase-dependent apoptosis is compromised, ROS may drive cell death via necroptosis. Necroptosis is a form of programmed necrosis characterized by necrotic morphology and dependent on the kinase activity of RIPK1 and RIPK3, leading to MLKL activation. Unlike apoptosis, which preserves membrane integrity, necroptosis is marked by pronounced cellular swelling and eventual rupture of the plasma membrane (Degterev et al., 2005; Zhang G. et al., 2019).

Studies have shown that shikonin exerts anticancer effects in the NPC 5-8F cell line by inducing necroptosis through increased ROS production and upregulation of receptor-interacting protein kinases 1 (RIPK1), RIPK3, and mixed lineage kinase domain-like pseudokinase (MLKL) (Liu et al., 2019). Shikonin-treated 5-8F cells exhibited ultrastructural features characterized by cytoplasmic translucency, loss of plasma membrane integrity, and preservation of nuclear membrane integrity. Inhibition of ROS by the antioxidant N-acetylcysteine (NAC) reduces the protein expression levels of RIPK1, RIPK3, and MLKL (Huang et al., 2013). In another study employing the CNE-2Z NPC cell line, shikonin was also found to promote necroptosis through the induction of ROS generation (Zhang Z. et al., 2017) (Figure 5).

## 5 Strategies and research progress in ROS-targeted therapy for NPC

### 5.1 Radiotherapy

Radiotherapy (RT) is the primary treatment modality for NPC, and its antitumor effects largely depend on ROS induced by ionizing radiation. Ionizing radiation can significantly increase intracellular ROS through mechanisms such as the ionization of water molecules and alterations in mitochondrial function, thereby inducing DNA double-strand breaks, mitochondrial damage, and cell death (Ding et al., 2023; Meng et al., 2020). Further studies indicate that changes in ROS levels during radiotherapy can influence tumor cell sensitivity to ionizing radiation. Antioxidants such as β-carotene, NAC, and GSH can suppress ROS and MAPK signaling, thereby exerting anti-apoptotic effects and potentially attenuating the antitumor efficacy of radiotherapy (Meng et al., 2020). Conversely, some antioxidant interventions may reduce treatment-related toxicity in normal tissues by limiting excessive oxidative damage (Rizkalla et al., 2026; Limbrunner et al., 2025). Therefore, the potential benefits and risks of concurrent antioxidant supplementation during radiotherapy remain controversial, and its clinical impact requires further investigation (Barnhart et al., 2024).

Clinical studies have begun to explore more selective redox-modulating strategies during radiotherapy. Avasopasem manganese, a superoxide dismutase mimetic, converts radiation-induced superoxide into H_2_O_2_ and O_2_ and has been shown to reduce severe oral mucositis in patients receiving chemoradiotherapy (Anderson et al., 2025). Two-year follow-up of an earlier randomized trial showed no significant differences in tumor-control outcomes between avasopasem and placebo (Anderson et al., 2022). In NPC, amifostine-mediated scavenging of radiation-induced free radicals also reduced radiotherapy-related mucositis without significantly affecting complete remission or survival outcomes in a multicenter randomized phase II trial (Chang et al., 2018).

Redox imbalance is one of the key factors contributing to radiotherapy resistance in NPC. The Nrf2/KEAP1 signaling pathway, a central regulatory axis of the antioxidant system, contributes to radiation resistance in NPC by reducing ROS (Zhou et al., 2020). Several molecules associated with oxidative stress, such as laminin subunit beta 3 (LAMB3), RASSF1A, and mitogen-activated protein kinase 15 (MAPK15), have also been shown to participate in the radiation response of NPC by regulating intracellular ROS levels (Zhuang et al., 2023; Si et al., 2023; Li Z. et al., 2018). ROS-related cell death processes also contribute significantly to the efficacy of radiotherapy. Ionizing radiation induces intracellular ROS accumulation and oxidative stress-related cell death, such as ferroptosis, which enhances antitumor effects (Yang et al., 2024; Zhou et al., 2025; Jin et al., 2025).

Furthermore, diverse microenvironmental and infection-related factors can influence radiotherapy efficacy by modulating ROS levels. *Fusobacterium* nucleatum infection, hypoxia, and EBV infection have all been reported to alter tumor-cell redox homeostasis and contribute to radioresistance (Chen H. et al., 2025; Hu et al., 2020; Guo et al., 2024). These findings provide a rationale for developing radiosensitization strategies targeting oxidative stress.

### 5.2 Chemotherapy

Chemotherapy remains an important component of the comprehensive treatment of NPC. The cytotoxic agents commonly used in clinical practice mainly include platinum-based agents and taxanes, and recent studies have confirmed that alterations in ROS levels are a core contributors to the cytotoxic effects of multiple chemotherapeutic agents.

Cisplatin is the most commonly used chemotherapeutic agent in NPC treatment, but its clinical use is limited by significant toxicities and the development of tumor resistance. Studies have shown that heme oxygenase-1 (HO-1) is upregulated in NPC, and it promotes cisplatin resistance in NPC cells by reducing oxidative stress levels and inhibiting ferroptosis (Cheng et al., 2025). To further improve therapeutic efficacy and reduce adverse effects, several next-generation platinum agents have been developed and introduced into clinical practice in recent years. Chen et al. found that lobaplatin, a third-generation platinum-based drug, exerts antitumor effects in NPC cells by inducing GSDME-mediated pyroptosis through ROS accumulation and JNK phosphorylation (Chen Z. et al., 2020). The clinical relevance of lobaplatin has been further supported by a recent multicenter randomized phase III trial with a median follow-up of 10.6 years, in which lobaplatin-based therapy achieved non-inferior 10-year progression-free survival compared with cisplatin-based therapy (70.7% vs. 71.9%), with lower rates of several cisplatin-associated late toxicities (Cao et al., 2026). However, despite advances in platinum-based chemotherapy, drug resistance remains a major challenge in NPC treatment. Nedaplatin has a more favorable toxicity profile than cisplatin, but cross-resistance between the two agents may occur. In cisplatin-resistant NPC cells, nedaplatin increases ROS levels and activates ERK signaling, thereby inducing cytoprotective autophagy and promoting cross-resistance (Liu et al., 2015). EBV infection can further reduce chemotherapy sensitivity by activating the p62–KEAP1–Nrf2 pathway, which upregulates GPX4 and decreases lipid ROS levels (Yuan et al., 2022).

During taxane therapy, Parkin overexpression enhances the sensitivity of NPC cells to paclitaxel through ROS-associated mitochondrial autophagy (Ni et al., 2023). PJA1 is highly expressed in tumors from NPC patients who are unresponsive to docetaxel, cisplatin, and 5-fluorouracil (TPF) chemotherapy, and its suppression of ROS-dependent GSDME-mediated pyroptosis contributes to docetaxel resistance (Huang et al., 2024). Furthermore, the deubiquitinating enzyme ubiquitin C-terminal hydrolase L1 (UCHL1) enhances tumor cell resistance to docetaxel by stabilizing caveolin-1 (CAV1) and suppressing lipid ROS production and ferroptosis. Erastin, a ferroptosis inducer, reduces docetaxel resistance associated with CAV1 overexpression (Ye et al., 2025).

### 5.3 Targeted therapy

Therapeutic strategies that disrupt redox homeostasis can suppress tumor growth and promote cell death in NPC. One major approach is to weaken the antioxidant defenses on which tumor cells depend to tolerate elevated oxidative stress. SLC7A11 is highly expressed in NPC, and its elevated expression is associated with poor prognosis, metastasis, and advanced T stage; it has been identified as an independent prognostic indicator. Targeting SLC7A11 limits GSH synthesis, leading to ROS accumulation, lipid peroxidation, ferroptosis, and apoptosis. Sorafenib, which inhibits SLC7A11 activity, also alters MAPK signaling, including p38 activation and ERK inhibition, and suppresses NPC growth in preclinical models (Wang et al., 2024). The clinical relevance of sorafenib in NPC has also been explored. In a phase II trial of recurrent or metastatic NPC, sorafenib combined with cisplatin and 5-fluorouracil achieved an objective response rate of 77.8% and a median progression-free survival of 7.2 months (Xue et al., 2013). More recently, N-acetyltransferase 10 (NAT10)-mediated upregulation of SLC7A11 was identified as a mechanism of sorafenib resistance by reducing ROS accumulation and ferroptosis, whereas NAT10 inhibition restored ferroptotic sensitivity in preclinical NPC models (Xue et al., 2024).

Other antioxidant systems also represent potential therapeutic vulnerabilities. PRDX3 knockdown suppresses the malignant behavior of NPC cells and induces mitochondrial dysfunction (Qiang et al., 2025),whereas SOD1 inhibition increases superoxide levels and lipid accumulation, thereby suppressing tumor cell growth (Liang et al., 2025). Targeting PBK similarly promotes ROS accumulation and activates JNK/p38 signaling, resulting in enhanced apoptosis and inhibition of NPC growth (Wang MY. et al., 2016).

The ROS production mechanism itself can also serve as a potential therapeutic target. Studies have found that the NOX inhibitor diphenyleneiodonium (DPI) exerts significant cytotoxic effects on LMP1-transformed and malignant cells, suggesting that targeting the NOX-ROS axis is a promising anticancer strategy (Sun et al., 2015). In addition to the above-mentioned molecular targeting strategies, emerging evidence suggests that natural products suppress NPC progression by modulating ROS homeostasis. A summary of representative studies is presented in Table 1. Overall, these findings highlight redox modulation as a promising strategy for NPC treatment.

**TABLE 1:** Natural products targeting ROS-related pathways in NPC.

Natural Products	Experimental Models	ROS regulation	Key Pathways	Anti-tumor effects	Reference
Allicin	HONE-1, HNE 1 cells	ROS↑	GSH/GPX4 axis	Induce ferroptosis	(Li et al., 2024c)
Solasodine	HONE-1, HNE1 cells,nude mouse xenograft model	ROS↑	HMOX1/GPX4 axis	Induce ferroptosis and inhibit tumor growth	(Wang et al., 2025b)
Isoquercitrin	CNE1, HNE1 cells, nude mouse xenograft model	ROS↑	AMPK/NF-κB p65 pathway	Induce ferroptosis and inhibit tumor growth	(Luo et al., 2024)
Berberine	S18,5-8F cells,nude mouse lung metastasis model	ROS↑	System Xc⁻/GSH/GPX4	Induce ferroptosis and inhibit metastasis	(Wu et al., 2024)
Brusatol	CNE2, 5-8F cells; nude mouse xenograft model	ROS↑	Nrf2/SLC7A11/GPX4 axis	Induce ferroptosis and act as a ferroptosis sensitizer	(Yang et al., 2026)
Lupeol	5-8F, CNE1 cells; nude mouse xenograft models	ROS↑	AMPK/NF-κB pathway	Induce ferroptosis and apoptosis	(Zhou et al., 2022)
Rosmanol	CNE2 cells	ROS↑	MAPKs/NF-κB pathway	Induce apoptosis	(Tao et al., 2025)
Apigenin	TW039, TW076 cells	ROS↑	ASM/PI3K/Akt /CDK1-cyclin B1 pathway	Induce apoptosis	(Chang et al., 2021)
Aloe-emodin	TW039, W076 cells	ROS↑	Caspase-8/Bid-tBid/Bax/Bcl-xL pathway	Induce apoptosis	(Lin et al., 2010)
Gambogenic acid	CNE1 cells	ROS↑	Akt/Bcl-2 family pathway	Induce apoptosis	(Yan et al., 2011)
AG4	CNE cells	ROS↑	Bcl-2 family/ caspase-9 and Fas/caspase-8 pathways	Induce apoptosis	(Dong et al., 2015)
Oridonin	CNE2Z, HNE1 cells	ROS↑	ROS-mediated mitochondrial pathway (Bcl-2/Mcl-1/Bax/caspase-3/PARP)	Induce apoptosis	(Zhang et al., 2017b)
Tetrandrine	TW076 cells	ROS↑	ER stress pathway (GRP780/ATF6/IRE1/GADD153/caspase-12)	Induce apoptosis	(Lin et al., 2016)
Bufalin	TW076 cells	ROS↑	Mitochondrial/ER stress/TRAIL pathways	Induce apoptosis	(Su et al., 2019)
Ophiopogonin B	C666- 1,HK 1 cells	ROS↑	Hippo–YAP/FOXO1 pathway	Induce apoptosis and inhibit proliferation	(Dong et al., 2021)
Naringin	TW039, W076 cells	ROS↑	Mitochondrial, death receptor, and ER stress pathways	Induce G1 cell-cycle arrest and apoptosis	(Chen et al., 2024)
Coronarin D	NPC-BM, NPC-039 cells	ROS↑	JNK/p38 MAPK pathway	Induce apoptosis and autophagy	(Chen et al., 2017)
Triptonide	HK-1 cells, 5-8F cells	ROS↑	p38 MAPK/mTOR/NF-κB pathway	Induce apoptosis and autophagy	(Geng et al., 2025)
Geraniin	C666- 1 cells	ROS↑	PI3K/Akt/mTOR pathway	Induce autophagy- mediated cell death	(Chen et al., 2022)
Shikonin	CNE2Z cells; nude mouse xenograft model	ROS↑	RIP1/RIP3-mediated necroptosis pathway	Induce necroptosis and inhibit tumor growth	(Zhang et al., 2017a)
Nobiletin	CNE-2, 5-8F cells; nude mouse xenograft model	ROS↑	KEAP1/NRF2/ ARE pathway	Induce apoptosis and inhibit proliferation, migration, and invasion	(Liang et al., 2025)
Resveratrol	TW01, TW06, HONE-1 cells; NPC CSC xenograft model in NOD/SCID mice	ROS↑	p53 signaling pathway	Inhibit stemness, EMT, and metabolic reprogramming	(Shen et al., 2013)

### 5.4 Immunotherapy

In recent years, immunotherapy has emerged as an important component of the comprehensive treatment of NPC. Existing research suggests that modulating ROS-related metabolic adaptation and restoring redox homeostasis in the tumor microenvironment may enhance the efficacy of immunotherapy.

Immune checkpoint inhibitors (ICIs) reactivate the antitumor immune system by targeting immune checkpoint pathways (Li et al., 2022; Wang et al., 2022; Grywalska et al., 2018). However, ICIs targeting PD-1 provide only limited benefits for patients with recurrent/metastatic nasopharyngeal carcinoma (RM-NPC). Chang et al. find that asparaginase induces metabolic adaptation and promotes ROS production. In parallel, asparagine deprivation enhances the antitumor effects of CD8^+^ T cells through ROS-mediated metabolic and signaling pathways. ROS-mediated modulation of T-cell differentiation promotes immune activation and enhances the efficacy of anti-PD-1 therapy, supporting the potential combination of asparaginase with anti-PD-1 therapy (Chang et al., 2025). In a recent compassionate-use study, L-asparaginase followed by pembrolizumab enhanced CD8^+^ T-cell fitness and antitumor responses in patients with anti-PD-1-refractory RM-NPC, providing preliminary clinical evidence that ROS-mediated metabolic modulation may enhance immune-checkpoint blockade (Chang et al., 2025). Another study demonstrated that cytokine-induced killer (CIK) cell therapy synergizes with anti-PD-1 therapy to exert antitumor effects by enhancing immune cell infiltration and alleviating oxidative stress in the tumor microenvironment (Chen et al., 2025b).

Overall, direct evidence regarding the role of ROS in NPC immunotherapy remains limited. However, existing studies suggest that ROS may participate in the regulation of immunotherapy efficacy by influencing T cell function and the tumor microenvironment. These findings offer a new direction for NPC immunotherapy research.

### 5.5 Emerging therapies

Emerging therapies based on ROS regulation are attracting increasing interest in NPC research. Photodynamic therapy (PDT) is an emerging ROS-based therapeutic strategy that has gained considerable interest in recent years. In a phase II study of patients with superficial residual or recurrent NPC after definitive radiotherapy, temoporfin-mediated PDT achieved a complete response in 20 of 21 patients (95%), with a 2-year local control rate of 75% and no serious adverse events (Stoker et al., 2015). Studies have shown that GdCPP, a multifunctional nanodiagnostic and therapeutic platform, can combine fluorescence imaging and magnetic resonance imaging (MRI) to achieve dual-modality imaging and generate large amounts of ROS under light exposure, thereby effectively killing NPC cells. GdCPP has demonstrated potent antitumor activity without significant damage to normal tissues (Yang et al., 2023). Hypericin-mediated photodynamic therapy (HY-PDT) induces excessive ROS, resulting in mitochondrial and DNA damage and ultimately promoting apoptosis in NPC cells (Wang et al., 2010). Furthermore, machine-learning-assisted screening has identified corrole as a potent photosensitizer that generates substantial ROS under irradiation and effectively eradicates cancer cells *in vitro* and *in vivo*. FLIM using this corrole enables effective discrimination between cancer cells and normal cells (Huang et al., 2025).

Nanomaterial-mediated ROS modulation is increasingly recognized as an emerging frontier in NPC treatment. Pan et al. found that Prussian blue nanozymes (PBNs) possess both catalase-like (CAT-like) and peroxidase-like (POD-like) activities. On the one hand, PBNs decompose H_2_O_2_ into O_2_, effectively alleviating the hypoxic tumor microenvironment. On the other hand, PBNs catalyze the conversion of H_2_O_2_ into ·OH, a type of ROS that, in excess, induces tumor cell apoptosis (Pan et al., 2024). Building on this, researchers further developed an α-lipoic acid-loaded hollow mesoporous Prussian blue nanozyme (AHPRzyme) for use in NPC mouse models. By depleting GSH and disrupting redox homeostasis, AHPRzyme sensitizes tumor cells to ROS-mediated cytotoxicity, culminating in apoptosis (Pan et al., 2025). More recently, a TfR1-targeted ferritin nanozyme (Pt-HFn) was developed to alleviate tumor hypoxia by catalyzing the conversion of H_2_O_2_ into O_2_, thereby enhancing NPC radiosensitivity. In NPC patient-derived xenograft models, Pt-HFn combined with radiotherapy significantly enhanced tumor suppression and prolonged median survival from 16 to 24 days (Zhang R. et al., 2025).

Gene therapy also offers a new research direction for NPC treatment. Researchers developed a ROS-responsive poly(β-amino ester) (PBAE) polymeric vector for delivering a CRISPR/Cas9 plasmid. This plasmid can specifically cleave LMP1, thereby inhibiting the growth of EBV-positive NPC cells (Yuan et al., 2024). Furthermore, bacterial vector-mediated PDT constitutes a promising therapeutic approach. In a nude mouse model, subcutaneous injection of KillerRed-expressing *Escherichia coli* (KR-E. coli) into CNE-2 tumor xenografts enabled bacterial proliferation within tumor tissue and ROS generation upon light exposure, ultimately inducing tumor necrosis without recurrence (Yan et al., 2015).

### 5.6 Combination therapy

Although monotherapy targeting ROS regulation has shown some promise in the treatment of NPC, it often fails to achieve the desired results because of the complex redox adaptation mechanisms of tumor cells. Consequently, in recent years, an increasing number of studies have explored combination-therapy strategies to modulate intracellular ROS levels in NPC cells, thereby enhancing therapeutic efficacy (Table 2).

**TABLE 2:** ROS-mediated combination therapeutic strategies for nasopharyngeal carcinoma.

Category	Main Drugs/Technology	Experimental Model	ROS-Related Mechanism	Reference
Radiotherapy-based combination strategies	DAC + radiotherapy	CNE2-RR and HONE1-RR cells; CNE2-RR nude mouse xenograft models	Activation of the TFAP2C-OTUD1-SLC25A11 axis increases ROS accumulation and apoptosis, thereby enhancing radiosensitivity and overcoming radioresistance	(Zhang et al., 2025a)
	Emodin + radiotherapy	CNE-1 cells, CNE-1 nude mouse xenograft models	Emodin enhances radiation-induced ROS generation; elevated ROS promotes apoptosis and suppresses HIF-1α expression, thereby enhancing radiosensitivity	(Hou et al., 2013)
	RuSe + X-ray irradiation	CNE-2 cells	X-ray-enhanced ROS overproduction induces mitochondrial membrane depolarization, sub-G1 arrest, and caspase-mediated apoptosis, thereby enhancing radiosensitivity	(Shi et al., 2023)
	Met-CuS@DSH + radiotherapy	HONE1 and C666-1 cells; HONE-1 nude mouse xenograft models	Metformin reduces mitochondrial O₂ consumption to alleviate hypoxia and enhance RT-induced ROS generation, while CuS depletes GSH to suppress ROS scavenging, thereby achieving bidirectional ROS amplification and enhancing radiosensitivity	(Chen et al., 2025)
	Artesunate + cisplatin	C666-1 and CNE2 cells; C666-1 and CNE2 SCID mouse xenograft models	Artesunate inhibits mitochondrial respiration, increasing mitochondrial superoxide and intracellular ROS and inducing oxidative stress; combined artesunate and cisplatin synergistically inhibit proliferation and induce apoptosis	(Li et al., 2017)
	Triptolide + cisplatin	HNE-1/DDP cells	Triptolide-induced ROS accumulation activates the mitochondrial apoptotic pathway, characterized by mitochondrial membrane depolarization, increased Bax and caspase-9, and decreased Bcl-2 and Mcl-1, thereby restoring cisplatin sensitivity	(Wang et al., 2015)
	hUC MSC-Exos + cisplatin	NPC-TW01 cells	Cotreatment elevates ROS, which acts upstream of Fas-mediated apoptosis, activating caspase-8, caspase-9, and caspase-3 and disrupting mitochondrial membrane potential, thereby enhancing cisplatin-induced apoptosis	(Li et al., 2025)
Chemotherapy-based combination strategies	Disulfiram/Cu + cisplatin	5-8F, CNE2, S18, and 6-10B cells; 5-8F BALB/c nude mouse xenograft models	DSF/Cu elevates cellular ROS to activate JNK/p38 MAPK signaling and apoptosis, while also inducing p53-mediated ferroptosis with increased lipid ROS; combination with cisplatin enhances antitumor efficacy *in vivo*	(Li et al., 2020)
	NEO212 + ganciclovir	C666.1 and patient-derived BM-NPC cells; C666.1 athymic nude mouse xenograft models	NEO212-induced ROS triggers ER stress and CHOP upregulation, thereby activating the EBV lytic cycle and caspase-mediated apoptosis; lytic induction sensitizes C666.1 cells to ganciclovir, resulting in synergistic cytotoxicity	(Hartman-Houstman et al., 2024)
	Bortezomib + SAHA	HK1-EBV, HONE1-EBV, HA, and C666-1 cells; C666-1, HONE1, and HA BALB/c nude mouse xenograft models	Bortezomib/SAHA synergistically increases ROS generation, which acts upstream of caspase activation to induce caspase-dependent apoptosis and suppress NPC growth	(Hui et al., 2013)
	Bortezomib + class I HDAC inhibitors (MS-275, apicidin, or romidepsin)	HA and C666-1 cells; HA BALB/c nude mouse xenograft models	Combination treatment induces ROS-dependent ER stress, with upregulation of ATF-4 and CHOP/GADD153, followed by caspase-4-initiated caspase activation and apoptosis	(Hui and Chiang, 2014)
	Mefloquine + Mcl-1 inhibitor (S64315)	HONE-1, HK-1, 6-10B, CNE-2, C666-1, 5-8F, HNE1, and CNE-1 cells; C666-1 subcutaneous xenograft mouse models	Mefloquine induces lysosome-mediated ROS generation and lipid peroxidation, while ROS also triggers compensatory Mcl-1 upregulation; pharmacologic Mcl-1 inhibition with S64315 counteracts this adaptive response, resulting in synergistic NPC cell death and sustained tumor growth suppression	(Dong et al., 2024)
Targeted therapy-based combination strategies	Brusatol + ferroptosis inducer (RSL3 *in vitro*; IKE *in vivo*)	CNE2 and 5-8F cells; 5-8F nude mouse xenograft models	Brusatol suppresses Nrf2 and its downstream antioxidant effectors SLC7A11, GPX4, and HMOX1, thereby enhancing ROS and lipid peroxidation and sensitizing NPC cells to ferroptosis induced by ferroptosis inducers	(Li et al., 2022)
	Celecoxib + RSL3	5-8F and CNE2 cells	Celecoxib elevates cellular ROS and upregulates the ferroptosis-related gene CHAC1, thereby sensitizing NPC cells to RSL3-induced ferroptotic cell death	(Li et al., 2026)
​	FA-MNP-CDDP-CBT	HNE-1 and HNE-1/DDP cells	Folate-mediated uptake and GSH-triggered intracellular aggregation increase nanoparticle retention and intracellular cisplatin concentration, while Fe_3_O_4_-mediated Fenton reactions amplify ROS generation, activating caspase-3/PARP-dependent apoptosis and reversing cisplatin resistance	(Bejjanki, et al., 2021a)
	GSH/ROS-responsive, mitochondria-targeted PEG-PLA@DIT-PAMAM (DOX/ICG; chemo/PDT/PTT)	HNE-1 cells, HNE-1 3D multicellular spheroids, and HNE-1 BALB/c nude mouse xenograft models	GSH-triggered nanocarrier disassembly promotes deep tumor penetration, while 808-nm laser-activated ICG generates ROS and heat; ROS cleaves thioketal linkers to trigger DOX release and induces mitochondrial damage and apoptotic signaling, thereby synergizing chemotherapy, PDT, and PTT	(Cheng et al., 2023)
	Lysosome-targeted, pH-responsive IRFEM@DHP (NIR-II imaging-guided PDT/PTT)	5-8F cells; 5-8F BALB/c nude mouse xenograft models	Acidic lysosomal conditions trigger IRFEM release and lysosomal accumulation; under 808-nm irradiation, IRFEM@DHP generates ROS and heat, causing lysosomal membrane permeabilization/rupture and lysosome-mediated cell death through synergistic PDT/PTT	(Li et al., 2024d)
Nanotechnology-driven combination strategies	pH-responsive charge-switchable ICG/Fe_3_O_4_/PA nanoclusters (PDT/PTT, ROS amplification)	HNE-1 cells; HNE-1 BALB/c nude mouse xenograft models	Tumor acidity triggers a positive surface-charge transition that enhances cellular uptake; under 808-nm irradiation, ICG-generated ^1^O_2_ is converted by palmitoyl ascorbic acid into H_2_O_2_, which undergoes Fe^2+^-mediated Fenton conversion to ·OH, thereby amplifying oxidative damage and synergizing PDT/PTT	(Bejjanki, et al., 2021b)
	GSH-responsive BTBT nanoparticles (PDT/PTT, multimodal imaging)	HONE1 cells; HONE1 nude mouse xenograft models	GSH-driven nanoparticle disassembly consumes intracellular GSH and relieves aggregation-caused ROS quenching, enhancing type-I ROS (O_2_•^-^/•OH) generation and photothermal conversion; the resulting redox imbalance, GPX4 suppression, lipid peroxidation, and mitochondrial damage activate caspase-mediated apoptosis	(Fang et al., 2025)
	NPC-targeted PtNCs-tipped AuNR nanoprobes (APINs; ICG/NTP -functionalized, NIR-II PTT/PECT/immunotherapy, multimodal imaging)	5-8F and CNE2 cells; NPC xenograft BALB/c mouse models	Under 1064-nm irradiation, AuNR-mediated LSPR enhances the POD-like catalytic activity of PtNCs, promoting H₂O₂ conversion to ·OH; the resulting photo-enhanced catalytic ROS generation synergizes with PTT to induce immunogenic cell death and activate antitumor immune responses	(Yang et al., 2026)

## 6 Discussion and conclusion

In conclusion, ROS in NPC act both as a drivers of tumor-promoting adaptation and as a crucial mediator through which various therapeutic approaches exert their antitumor effects. The underlying mechanism is that tumor cells maintain oxidative stress within a dynamic steady-state range that is “survival-promoting yet non-lethal” through coordinated antioxidant defenses, metabolic remodeling, and mitochondrial quality-control mechanisms. Within this adaptive range, ROS can support tumor cell survival, metabolic reprogramming, immune regulation, and treatment resistance, whereas excessive ROS levels that exceed the cellular buffering capacity may trigger autophagy-associated stress responses or regulated cell death. Therefore, the “double-edged sword” nature of ROS determines the complexity of translating ROS targeted strategies into clinical practice. Simply increasing or inhibiting ROS is unlikely to produce consistent therapeutic benefits, because the biological outcome of ROS modulation depends not only on ROS intensity, also on cellular context, antioxidant capacity, and the specific stress-response or cell death pathways involved. The key challenge is therefore to precisely regulate the magnitude, timing, and spatial distribution of oxidative stress while selectively disrupting the redox adaptations of tumor cells.

Another major challenge is tumor heterogeneity. The viral background, metabolic state, antioxidant capacity, and treatment history of NPC may generate distinct redox dependencies among patients. EBV-associated NPC has relatively well-defined links between viral proteins and ROS dysregulation, whereas the mechanisms underlying ROS imbalance in EBV-negative and HPV-associated NPC remain less well characterized. This heterogeneity complicates the identification of a universally effective ROS-targeted strategy. At the same time, enhancing ROS-mediated antitumor effects while avoiding oxidative damage to normal tissues remains a critical issue, particularly in the context of radiotherapy and combination treatment.

From a translational perspective, the current evidence is also uneven. Although several ROS-associated therapeutic approaches have entered clinical evaluation and recent studies have provided encouraging efficacy data, many strategies that directly target redox regulators, ROS-dependent cell-death pathways, or ROS-responsive nanoplatforms remain predominantly supported by *in vitro* and animal studies. In addition, adaptive antioxidant and stress-response mechanisms may allow tumor cells to restore redox homeostasis after treatment, thereby contributing to therapeutic resistance. This may partly explain why ROS-targeted monotherapy often shows limited efficacy and why rational combination strategies are increasingly being explored. Overall, a deeper understanding of ROS homeostasis and redox adaptation in NPC may help guide the development of more precise and effective therapeutic strategies.

## Glossary

AMPK: AMP-activated protein kinase
CAT: Catalase
CRISPR/Cas9: Clustered regularly interspaced short palindromic repeats/CRISPR-associated protein 9
CSCs: Cancer stem cells
CTLs: Cytotoxic T lymphocytes
DAC: Decitabine
EBNA1: Epstein–Barr nuclear antigen 1
EBV: Epstein–Barr virus
ER: Endoplasmic reticulum
ETC: Electron transport chain
FOXO3: Forkhead box O3
GPX: Glutathione peroxidase
GPX4: Glutathione peroxidase 4
GSDMD: Gasdermin D
GSDME: Gasdermin E
GSH: Reduced glutathione
GSSG: Oxidized glutathione
HDAC: Histone deacetylase
HIF-1α: Hypoxia-inducible factor 1α
HPV: Human papillomavirus
ICIs: Immune checkpoint inhibitors
KEAP1: Kelch-like ECH-associated protein 1
LDHA: Lactate dehydrogenase A
LMP1: Latent membrane protein 1
lncRNA: Long non-coding RNA
MAPK: Mitogen-activated protein kinase
miRNA: MicroRNA
MLKL: Mixed lineage kinase domain-like pseudokinase
mTOR: Mechanistic target of rapamycin
NADPH: Nicotinamide adenine dinucleotide phosphate
NF-κB: Nuclear factor kappa-B
NLRP3: NOD-like receptor family pyrin domain-containing 3
NOX: NADPH oxidase
NPC: Nasopharyngeal carcinoma
Nrf2: Nuclear factor erythroid 2-related factor 2
OXPHOS: Oxidative phosphorylation
PD-1: Programmed cell death protein 1
PDT: Photodynamic therapy
PRDX: Peroxiredoxin
RIPK1: Receptor-interacting protein kinase 1
RIPK3: Receptor-interacting protein kinase 3
RM-NPC: Recurrent/metastatic nasopharyngeal carcinoma
ROS: Reactive oxygen species
RT: Radiotherapy
SOD: Superoxide dismutase
TCA: Tricarboxylic acid
TIGAR: TP53-induced glycolysis and apoptosis regulator
TME: Tumor microenvironment
UPR: Unfolded protein response
